# A Statistical Memoir on the Influence of Various Professions on the Health and Mortality of Mechanics and Artisans in the Prime of Life; Founded on the Tables of the Institution for Sick Mechanics in Würtzburg from 1786 to 1834

**Published:** 1840-10

**Authors:** 


### Art. IV.

*Ueber den Einfluss der verschiedenen Gewerbe auf den Gesundheitszustand und die Mortalität der Künstler und Handwerker in den Blüthenjahren, &c.* Von Dr. C. H. Fuchs.

*A Statistical Memoir on the Influence of Various Professions on the Health and Mortality of Mechanics and Artisans in the prime of life; founded on the Tables of the Institution for Sick Mechanics in Würtzburg from* 1786 to 1834. By Dr. C. H. Fuchs, Professor of Medicine in Wurtzburg.—*Berlin*, 1835. 8vo.

Professions in medical statistics may be considered certain determined combinations of external circumstances to which classes of men are exposed. Their influence is much more powerful and obvious in India, where they are hereditary, than in Europe; yet several professions in Europe have a remarkable effect in modifying the extent of sickness and death to which their members are liable. Before this full effect can be determined, extended series of observations, made by different individuals, must be collected; and the observations must embrace all essential facts, capable, like other facts in physical science, of being compared. To determine the relative health of any profession or class of men, and the deleterious influence of any circumstance in which they may be placed, the facts required are simple and few; but it is very rare that they can all be obtained or at least are all furnished by writers. In the first instance it is necessary to ascertain the number of individuals in each profession between the ages of 10-20, 20-30, &c., and then the total cases of disease, the average number constantly sick or the days of sickness, and the deaths in a determined time; from which data the annual diseases and deaths in 1000, and the average proportion of sick at all ages may be deduced. The sicktime and mortality in an assumed unit of lifetime thus becomes a determined quantity, which may be compared with any other quantity. To give an example: in the Island of Corfu* the average force of the British troops was 1974·5 during the six years 1816-21; equivalent to 11,847 (1974·5 × 6) years of life, 620 of which were years of sickness. The total number of cases treated amounted to 14,098. Whence it is found by direct proportion that in a year, 1000 soldiers living suffered 1190 attacks of sickness; that 52 of them were on an average constantly ill, and that 27 died. Of 1000 cases treated, 23 terminated fatally. A table in which these results were compared with those presented by other classes of men, would constitute a law of of health for professions: it would teach the medical practitioner how many patients in a known population he may expect to attend; the general on how many effective men he may count in a campaign; the government how the labouring, manufacturing, agricultural, and higher classes are affected by political or economical measures. The sick-clubs and self-supporting dispensaries can only be firmly established by a knowledge of the quantity of sickness in the class they embrace; and if they enter every individual, distinguishing the age on their books, they will soon be in possession of a mass of useful facts.

The observations of Professor Fuchs were collected from the reports

* Hennen's Medical Topography of the Mediterranean. We have not been fortunate enough to meet with all these data in any other work foreign or English. This article was written before the appearance of the army medical reports.

of a Trades' Institution in Würtzburg, to which each workman employed contributed weekly, in order to obtain a certain sum in sickness. These reports, published since 1788 distinguish the contributions of each trade; the deaths and attacks of sickness occurring in each trade have been distinguished from the first: and from these contributions and payments the learned Professor has been enabled to ascertain that since the foundation of the society in 1786 (49 years), the average number of contributions has been 1186; the same as if 58,125 members had lived and been occupied in their respective arts one year in Würtzburg: 275 cases of sickness and 9 deaths occurred on an average annually; for the total attacks of sickness in 49 years amounted to 13,268; the deaths to 445. Of 10,000 contributors 2282 fell sick and 76 died yearly; and of 10,000 cases treated 327 (1 in 29·8) ended fatally. The value and defect of Professor Fuchs's method of enquiry are sufficiently obvious; but we shall compare it with what was said before to impress the right method more forcibly on future observers.

| Professions. | Mean Age. | In 1000 living, the ANNUAL | | | Deaths in 1000 cases of sickness. |
|---|---|---|---|---|---|
| | | Cases of Disease. | Number constantly sick. | Deaths | |
| Soldiers, &c. (in Corfu). | Nearly 26 | 1190 | 52·34 | 27·4 | 23·05 |
| Artisans, &c. (in Wurtzburg). | 15-35 | 228·2 | | 7·6 | 32·70 |

The ages of neither class were certainly known; and this table only shows the attacks of sickness and deaths at the ages specified; the attacks of sickness are however probably the same at all ages between 10-70. A material defect in the German Professor's researches, it will be perceived, is the omission of the average proportion of sickness: investigated in the friendly societies of Great Britain by Dr. Price and the Highland Society, a defect which the data already before him will probably supply. It is almost unnecessary to remark that the excessive mortality and sickness of the soldiers is in part owing to the climate and fevers of the Mediterranean. The mortality and sickness of soldiers in times of peace is in their native climate greatly above the average; of the English infantry 18 in a thousand die annually, while the mortality of the entire population is only 10 in a thousand at the corresponding age. The mortality of the Würtzburg artisans is apparently low; Dr. Fuchs states that the ages of the different classes were nearly the same, on an average between 15 and 35 years, and this may be fairly brought within the narrower mean of 20-30; at which period of life the mortality in Carlisle was 7·54 per 1000, differing to a trifling extent from 7·60. With Dr. Fuchs we are disposed to place considerable reliance on this general result, although the body of artisans in the society were to a certain extent selected; and we subjoin for comparison the deaths in 1000 at the same age in England and Wales, in Belgium, and in Sweden:

| England and Wales. | Belgium. | Sweden. | Würtzburg. | Carlisle. |
|---|---|---|---|---|
| 10·1 | 9·1 | 8·9 | 7·6 | 7·5 |

The proportion of 32·7 deaths in 10,000 cases treated, the Professor thinks highly favorable; but on what grounds we do not know. Nearly the same mortality (33·4) has been observed among the sick of the British troops in Bengal;* and in Corfu, only 230 died in 10,000 treated. At the same time the attacks of sickness suffered by 1000 persons in Würtzburg, Corfu, and Bengal, were in the proportion of 228; 1190; 1713.

The mean mortality of the Würtzburg artisans, expressed in parts of 10,000, being 76; the mean frequency of sickness, 2282; when the same operation has been repeated for each profession, on a sufficient number of individuals, their relative *mortality* and what Professor Fuchs has called their *morbility* may be determined by comparison with the mean standard.

Before examining the professions separately, it is necessary to observe that: 1. The institution, and consequently the calculations, only embrace the incorporated trades; day labourers, hackney coachmen, and some others are consequently excluded. 2. That only journeymen and apprentices belong to the institution. And this is so far for the advantage of the calculation, as the sick are nearly of the same age (15-35); besides, for obvious reasons, a profession exercises its baneful influence far more intensely on journeymen and apprentices than on their masters, who often only superintend without working at their business, and so avoid its discomforts and dangers. 3. Formerly several trades, for example, watchmakers and gunsmiths, carpenters and slaters, &c. formed one united company, and were placed together in the accounts. Professor Fuchs had no means of separating these associated trades, and was himself compelled to combine several of the smaller allied trades, in order to obtain more probable results. 4. Members having syphilitic diseases, and, up to the year 1828, also itch, were not entered on the books.

The second and third columns of the following tables, showing the total deaths and diseases of each profession, were calculated from the payments made in the 49 years; but the contributors belonging to each profession were only known from 1818 to 1834; and the numbers in the tables were obtained by first ascertaining the relation of the sick to the contributors during these 17 years, and calculating from the relation so obtained the number of contributors in the earlier period. For example, of 920 weavers who contributed between 1818 and 1834, exactly 269 had an attack of sickness; and, as during the 49 years 480 of the weavers fell ill, the following proportion gives the total number of weavers who had contributed, 269 : 920 : : 480 : 1642; which last sum is entered in the first column of the table.

We purposely withhold the arrangement of these 54 trades in the elaborate table of Professor Fuchs, according to their greater or less mortality and morbility, and all the Professor's reasonings on their relative salubrity. Professor Fuchs is apparently ignorant or forgetful of the well-established physiological fact that the fluctuations of mortality between 15 and 35 are limited to nearly 7-20 in 1000 of those who remain in their native climate, or he would not reason seriously with the great anomalies of the fifth column before his eyes. These anomalies are principally due to the paucity of observations, which may be sufficiently augmented if proper returns were obtained from all similar institutions; on this account the

* Burke on the Mortality of Troops in India, 1826-32. Edinb. Journal, No. 109.

paper before us deserves attention. It is one of the numerous series of observations which we hope to collect in this Journal, from which our readers, or perhaps ourselves at some future time, may deduce rational, well-founded generalizations. It may, however, be observed, that in this arrangement and in that which follows, those classes in which the morbility exceeds 2282, the mortality, 76, are considered unhealthy; on the contrary, those professions are considered salubrious in which the proportions fall below the average.

The influence of a profession on health is the result of several appreciable agents, to determine the exact value of which is the next step in the enquiry. For this purpose Dr. Fuchs has grouped all the professions in the table according to the locality in which they are carried on; the temperature and moisture connected with their exercise; the materials employed; the muscular force required; the position and necessary movements of the body; the aliment insured to their followers; the amount of wages; their influence on the education and the state of mind of their members. This has the further advantage of increasing the number of facts in each category, and diminishing the chance of error from differences of age. The first column shows the extent of the observations or the number of yearly contributors in the group; the second the attack of sickness in 10,000 persons living one year; the third, the annual deaths in 10,000; and the fourth the deaths in 10,000 cases treated. It is right to observe that the morbility is diminished disproportionably in some professions, where the contributors were well off, and resorted less frequently than others to the institution; all the deaths were recorded. To catch the eye more readily, an * has been affixed where the mortality and morbility were above, a † where they were below the average; the * therefore indicates unhealthy professions when over the column of morbility and mortality.

| INFLUENCE OF LOCALITY. | Total Contributors. | Morbility. | Mortality. | Relative Mortality. |
|---|---|---|---|---|
| *Professions carried on* (1) *in the Open Air.* | 5622 | †2138 | *99 | 465 |
| Thatchers, fishermen, sailors, gardeners, masons, stonecutters, plasterers, carpenters, and slaters. | | | | |
| [The increased mortality is probably owing to accidents.] | | | | |
| (2) *Alternately in the Open Air and Indoors.* | 11700 | †2212 | †59 | 266 |
| Coopers, surgeons, hairdressers, glaziers, farriers, chimneysweepers, butchers, millers, tanners, grinders, ropemakers, waxchandlers, wheelwrights. | | | | |
| (3) *in Confined Rooms.* | 40793 | *2341 | *78 | 338 |
| The 34 remaining professions. | | | | |
| (4) *partly in Water.* | 3569 | *2536 | †70 | 276 |
| Fishermen, sailors, millers, tanners. | | | | |
| INFLUENCE OF TEMPERATURE AND MOISTURE. | | | | |
| *Professions carried on* (1) *in Warm, Dry Air.* | 660 | *2892 | †66 | 228 |
| Blacksmiths, coppersmiths, locksmiths, nailers, goldsmiths, beltmakers, chimneysweepers, swordmakers, tinmen, artillery-smiths, pewterers. | | | | |

| | Total Contributors. | Morbility. | Mortality. | Relative Mortality. |
|---|---|---|---|---|
| (2) *in Warm, Moist Air.* Bakers, brewers, confectioners, potters, soapboilers, gilders, lackerers, waxchandlers. | 6877 | †1913 | †52 | 273 |
| (3) *in Cold, Moist Air.* Printers, dyers, grinders, plasterers, weavers, and the professions carried on in water. | 8405 | †2165 | *83 | 384 |
| (4) *in Common Air.* The 27 remaining professions. | 36183 | †2268 | *81 | 359 |
| (5) *in an Elevated Temperature.* | | *2393 | †59 | 247 |
| (6) *in Moist Air.* | | †2051 | †69 | 338 |
| (7) *in Rapid Alternations of Heat and Cold.* Blacksmiths, barbers, hairdressers, chimney-sweepers, brewers, millers, fishermen, and tanners. | 7093 | *2482 | 86 | 346 |
| INFLUENCE OF THE MATERIALS EMPLOYED. | | | | |
| *Professions carried on* (1) *in a Dusty Atmosphere.* | 18602 | †2267 | †62 | 275 |
| (2) *in an Atmosphere of Mineral Particles.* Stonecutters, masons, plasterers, locksmiths, blacksmiths, artillery-smiths. | 7827 | *2762 | *77 | 311 |
| (3) *in an Atmosphere of Vegetable Particles.* Bakers, millers, hairdressers, chimney-sweepers, ropemakers. | 8174 | †2160 | †59 | 278 |
| (4) *in an Atmosphere of Animal Particles.* Saddlers, furriers, tapesterers, brushmakers, clothbearers, hatters. | 2556 | †1878 | †23 | 125 |
| (5) *in Smoke and Carbonaceous Vapours.* Blacksmiths, locksmiths, nailers, sword-makers, tinmen, artillery-smiths, pewterers, coppersmiths. | 5342 | *3167 | †71 | 224 |
| (6) *in Emanations of Mercury, Lead, and Arsenic.** Pewterers, coppersmiths, tinmen, goldsmiths, beltmakers, hatters, potters, plasterers. | 4732 | 1525 | 58 | 388 |
| (7) *in Metallic Emanations generally.* | | 2430 | 63 | 263 |
| (8) *in Vegetable Emanations.* Bakers, coopers, brewers, dyers, lackerers, weavers. | 9542 | 2202 | 61 | 276 |
| (9) *in Animal Exhalations.* Butchers, tanners, soapboilers, waxchandlers. | 3768 | 1759 | 32 | 181 |

* The greater part of these trades are conducted on a very small scale at Würtzburg; and many precautions are taken against the deleterious influence of metallic vapours.

| | Total Contributors. | Morbility. | Mortality. | Relative Mortality. |
|---|---|---|---|---|
| INFLUENCES OF MUSCULAR EXERTION. | | | | |
| *Professions requiring* (1) *great Muscular Efforts.* | 13383 | †2133 | †67 | 317 |
| Brewers, coopers, thatchers, sailors, blacksmiths, masons, stonecutters, butchers, millers, nailers, wheelwrights, carpenters, slaters. | | | | |
| *Professions in which* (2) *but moderate exertion is required.* | 18926 | *2371 | *104 | 439 |
| Glovers, lacemakers, bookbinders, brushmakers, surgeons, confectioners, dyers, hairdressers, goldsmiths, shopkeepers, farriers, tailors, shoemakers, tapesterers, watchmakers, gilders, pewterers. | | | | |
| (3) *Slight, but continued Activity is required.* | 26316 | †2211 | †58 | 264 |
| The 36 remaining professions. | | | | |
| INFLUENCES OF CERTAIN DETERMINED POSITIONS AND MOVEMENTS. | | | | |
| *Professions in which the Men* (1) *are almost always Sitting.* | 18829 | *2577 | *99 | 385 |
| Glovers, lacemakers, brushmakers, goldsmiths, beltmakers, furriers, sadlers, tailers, shoemakers, tinmen, weavers, watchmakers. | | | | |
| (2) *Generally Stand at work.* | 22648 | *2451 | †69 | 281 |
| Bakers, bookbinders, dyers, glaziers, blacksmiths, turners, coppersmiths, butchers, nailers, grinders, locksmiths, joiners, swordmakers, ropemakers, plasterers, wheelwrights, carpenters, artillery-smiths. | | | | |
| (3) *Alternately Sit and Stand.* | 16648 | †1713 | †61 | 357 |
| All the other professions. | | | | |
| (4) *Work in a Stooping Position.* | 24943 | *2858 | *95 | 336 |
| *Sitting*—glovers, brushmakers, goldsmiths, tailers, shoemakers, watchmakers. *Standing*—glaziers, grinders, locksmiths, joiners, wheelwrights, gilders, pewterers, coopers, gardeners, tanners. | | | | |
| (5) *Carry Burdens.* | | †1939 | †69 | 355 |
| (6) *Climb, go up and down Stairs.* | | †2100 | †74 | 355 |
| (7) *Use the Axe or Hammer.* | | †2211 | *81 | 368 |
| (8) *Work with the Four Extremities.* | | †2220 | †71 | 318 |
| INFLUENCES OF ALIMENTS. | | | | |
| *Professions which prepare* (1) *Articles of Food.* | 6983 | †1732 | †38 | 223 |
| Bakers, butchers, gardeners, confectioners. | | | | |
| (2) *Drinks.* | | *2378 | †63 | 266 |
| Brewers, coopers. | | | | |

| Influences of Wages. | Total Contributors. | Morbility. | Mortality. | Relative Mortality. |
|---|---|---|---|---|
| *Professions* (1) *paid well in Summer, badly in Winter.* | | †2138 | *99 | 465 |
| Thatchers, fishermen, gardeners, masons, plasterers, carpenters. | | | | |
| (2) *which obtain High Wages.* | 9886 | †1634 | †59 | 365 |
| Brewers, lacemakers, printers, glaziers, goldsmiths, beltmakers, shopkeepers, tanners, swordmakers, ropemakers, tapesterers, watch and gun makers, gilders. | | | | |
| (3) *which gain Low Wages.* | 19858 | *2714 | *103 | 380 |
| Glovers, brushmakers, barbers, hairdressers, potters, blacksmiths, butchers, nailers, grinders, tailers, shoemakers, weavers, coopers (Weissküffner.) | | | | |
| (4) *which gain Moderate Wages.* | 22759 | †2223 | †59 | 247 |
| The remaining 21 professions. | | | | |
| Influence of Education, and State of Mind. | | | | |
| *Professions of which the common Members* (1) *receive a tolerable Education.* | 4928 | †1118 | †73 | 653 |
| Shopkeepers, printers, goldsmiths, sculptors, watchmakers, &c. | | | | |
| (2) *can scarcely hope to become Masters.* | 9360 | †1929 | *86 | 448 |
| Brewers, printers, millers, masons, carpenters. | | | | |
| (3) *are subject to Reverse of Fortune.* | | | | |

Under this last head are to be classed those professions which depend for their existence on commerce, or in small German towns, such as Würtzburg, principally on fashion. "The most striking example," says Dr. Fuchs, "of the influence exercised by fashion on the health of a profession, is furnished by the hairdressers of Würtzburg. The 22 masters who, with numerous journeymen, in 1803, dressed the hair and the wigs of the inhabitants of Würtzburg, have declined to 8, who together keep but 5 men, and the profession which Adelmann esteemed healthy and ever cheerful, has lost its high spirits, and occupies, by its great mortality (196), one of the lowest places among the unhealthy professions. I am much inclined to ascribe the unfavorable state of health experienced by the hairdressers in great part to the mental impression which the decline of that noble art has exercised on its disciples."

In stating that the increased mortality of the hairdressers was, in our opinion, *chiefly* owing to the greater proportion of *old men*—to a necessary difference of age, such as is found in all declining professions—we do not wish to cast a doubt on the great sensibility of those distinguished artists who have so long decorated the heads of the inhabitants of Würtzburg. No doubt the sudden fluctuations of wages and employment in the manufacturing towns of this kingdom have a most powerful effect on the health and mortality of the operatives.

To sum up the results of this investigation, in reply to the question—"What influence have professions on the morbility and mortality, independently of the form of malady; what professions are healthy, what unhealthy, and why are they so?" Dr. Fuchs lays down these inferences:

1. The influence of professions on the morbility and mortality in general is remarkable; the difference in these respects between particular professions are great.

2. The mortality and morbility do not always proceed in the same ratio. Many professions have great numbers sick and few deaths—and the reverse: only 8 professions (see Table) are equally distinguished by a high morbility and relative mortality; and 16 others have few sick and proportionally few deaths.

3. According to the absolute *mortality*—according to the loss which a given number of individuals belonging to the same profession suffer in a year—22 professions are unhealthy, 34 are healthy.

4. Some agencies regulate the frequency of sickness (morbility), others the fatality of diseases (relative mortality); and hence it is clear that in particular professions the number of sick, while in others the number of deaths, will be high, without the one invariably answering to the other.

5. The absolute mortality, on the contrary, is only influenced by causes which either uniformly raise or lower the morbility and relative mortality, or exercise a *preponderating influence* on one of them; and is therefore, although its oscillations are generally of a more limited extent, the surest measure of the salubrity of professions.

The results of the present investigations appears then to indicate that the following circumstances connected with professions augment the mortality:—slight muscular exercise, bad wages, constant sitting, the bent posture, exposure to changes of temperature, low spirits, cold, moist air, cold dry air, working in-doors, mineral dust. They confirm the conclusions of Dr. Lombard with regard to the unfavorable influence of penury and misery on health, one of the best-established principles in statistics. A sedentary life is shown by each series of observations to be injurious. That education promotes health is, on the contrary, established by both. With regard to the influence of dusts and vapours the results differ; in fact, neither possessed the means of deciding this question.

This memoir displays great industry, accuracy, and considerable acquaintance with statistics; in many respects it merits more attention than anything that has yet been written on the hygiène of professions. One capital fact it establishes that in favorable circumstances 1 man in 4, or 22·8 per cent. has an attack of sickness annually; or, more precisely, 23 per cent. apply to friendly societies on the ground of sickness. Similar and more comprehensive researches we hope soon to see instituted in every part of Great Britain where there are Benefit and Health Assurance Societies.

